# Congenital Heart Disease Fetuses Have Decreased Mid-Gestational Placental Flow, Placental Malperfusion Defects, and Impaired Growth

**DOI:** 10.1016/j.jacadv.2024.101559

**Published:** 2025-01-16

**Authors:** Rebecca Josowitz, Deborah Y. Ho, Somya Shankar, Antara Mondal, Alexis Zavez, Rebecca L. Linn, Zhiyun Tian, J. William Gaynor, Jack Rychik

**Affiliations:** a Division of Cardiology, Children’s Hospital of Philadelphia, Philadelphia, Pennsylvania, USA; b Division of Pediatric Cardiology, Stanford School of Medicine, Lucile Packard Children’s Hospital, Palo Alto, California, USA; c Department of Biomedical and Health Informatics, Children’s Hospital of Philadelphia, Philadelphia, Pennsylvania, USA; d Division of Anatomic Pathology, Department of Pathology and Laboratory Medicine, Children’s Hospital of Philadelphia, Perelman School of Medicine, University of Pennsylvania, Philadelphia, Pennsylvania, USA; e Division of Cardiothoracic Surgery, Department of Surgery, Children’s Hospital of Philadelphia, Perelman School of Medicine, University of Pennsylvania, Philadelphia, Pennsylvania, USA; f Department of Pediatrics, Perelman School of Medicine at University of Pennsylvania, Philadelphia, Pennsylvania, USA

**Keywords:** congenital heart disease, fetal, malperfusion, placenta

## Abstract

**Background:**

Placental health may impact the development and outcomes of congenital heart disease (CHD). CHD fetuses have been shown retrospectively to have decreased placental blood flow.

**Objectives:**

The purpose of this study was to determine if CHD fetuses with decreased placental blood flow have placental pathology at birth and if there is a relationship between placental blood flow, placental pathology, and outcomes.

**Methods:**

We performed a prospective case-control study of 38 CHD fetuses, including 28 with single ventricle physiology and 36 controls. Demographic, clinical, and postnatal biometric data were collected. Umbilical venous volume flow (UVVF) was measured from 2nd trimester fetal echocardiograms. Placentas underwent standardized pathological analysis. Standard descriptive statistics and regression analyses were performed to analyze the relationship between UVVF, placental defects, and outcomes.

**Results:**

CHD fetuses had a 15% decrease in mid-gestational UVVF indexed to fetal weight (*P* < 0.01), and a 27% reduction in UVVF as a proportion of fetal cardiac output (*P* < 0.01) compared to controls. CHD fetuses had increased placental maternal vascular malperfusion (MVM) lesions (44% vs 18%, *P* < 0.05), especially high-grade MVM (39% vs 9.1%, *P* = 0.05), and a trend toward increased placental fetal vascular malperfusion lesions (42% vs 23%, *P* = 0.10). Placental MVM but not fetal vascular malperfusion lesions were associated with decreased birth weight in CHD fetuses (*P* < 0.001). There was no association between UVVF and placental pathologic findings or fetal growth.

**Conclusions:**

CHD (particularly single ventricle) fetuses have decreased mid-gestational placental blood flow, increased placental malperfusion defects, and impaired fetal growth. Placental MVM may influence impaired fetal growth in CHD.

The majority of congenital heart disease (CHD) remains unexplained by currently identified genetic or environmental factors. The maternal-fetal environment may impact the development of, and outcomes associated with, CHD.^1^, ^2^, ^3^ Our understanding of the role of the placenta, despite its critical place in the uterine environment, is quite limited.

The fetal heart and placenta develop concurrently and share several key developmental pathways.^1^ Placental and umbilical cord abnormalities are associated with an increased risk of fetal CHD.^4^, ^5^, ^6^ Likewise, there is a known association between maternal hypertensive disorders of pregnancy and fetal CHD.^7^^,^^8^ Specifically, abnormal vascular development and an imbalance between circulating angiogenic and antiangiogenic factors is implicated in the pathophysiology of both placental abnormalities and CHD.^9^^,^^10^

Our limited understanding of the placenta in CHD is in part due to the challenges of assessing the organ in utero. In CHD, traditional Doppler indices of placental blood flow such as umbilical artery (UA) flow and the cerebroplacental ratio (CPR) can vary considerably^6^^,^^11^^,^^12^ and are often normal.^13^^,^^14^ Umbilical venous volume flow (UVVF) is a validated noninvasive Doppler-derived method to directly assess blood flow from placenta to fetus.^15^ UVVF measurements are highly reproducible; however, additional studies are required to standardize the methodology.^16^ UVVF is an early indicator of fetal growth restriction and uniquely reflects characteristics of the placenta rather than the fetus.^17^^,^^18^ We previously demonstrated decreased mid-gestational UVVF parameters in CHD fetuses compared to controls.^19^ In this work, we sought to determine whether CHD fetuses with impaired placental blood flow demonstrate distinct pathological features of the placenta at birth and explore their relationship with adverse clinical outcomes.

## Methods

### Study design

This was a single-center prospective case-control study performed in the Children’s Hospital of Philadelphia’s (CHOP) Fetal Heart Program and approved by the CHOP Institutional Review Board (IRB# 17-014630). Patients were consecutively enrolled from February 2018 to October 2019. Maternal-fetal dyads who obtained a fetal echocardiogram at CHOP’s Fetal Heart Program either for clinically indicated screening or follow-up for CHD were eligible to participate in the study. Inclusion criteria for controls were fetal gestational age of 18 to 28 weeks, no structural heart disease on fetal echocardiogram, and consent to transfer the placenta to CHOP after delivery. Exclusion criteria for all subjects were multiple gestation, major extracardiac congenital abnormalities (such as congenital diaphragmatic hernia), significant fetal arrhythmia, hydrops fetalis, and the presence of hemodynamically significant maternal CHD (moderate or greater complexity).^20^ All eligible dyads were approached for study participation. Enrolled fetuses with CHD included those with single ventricle (SV) CHD (n = 28), tetralogy of Fallot (n = 9), and D-transposition of the great arteries (n = 1). Thirty-six control fetuses were enrolled. Mothers of 11 cases were simultaneously enrolled in a prospective randomized clinical trial of vaginal natural progesterone therapy vs placebo (NCT02133573) to evaluate the effect on neurodevelopment for fetuses with CHD. Placebo or progesterone was administered twice daily between 28 and 39 weeks gestation. Randomization of overlapping study subjects remained blinded to this study’s investigators.

The majority of SV CHD were single right ventricle lesions, including hypoplastic left heart syndrome (n = 24) and its variants, such as double outlet right ventricle with mitral atresia (n = 2), and severely right-dominant unbalanced complete atrioventricular canal defects (n = 1). One patient with tricuspid atresia and unobstructed normally related great arteries was included in the SV category. All cases underwent prenatal and/or postnatal genetic evaluation.

Fetal echocardiograms were performed via standard protocol which included assessment of fetal biometry, umbilical vein and artery Doppler tracings, and cardiac rhythm and anatomy. Echocardiographic findings from 2nd trimester studies were compared between cases and controls, however all follow-up fetal echocardiograms performed for cases were included to evaluate longitudinal changes in UVVF. Measurements determined to be inaccurate due to inadequate 2nd trimester image quality were excluded. Controls had single studies performed with no follow-up. Other requisite data collection prior to delivery occurred via medical record review and no additional prenatal visits were required. Medical record review included maternal demographics, fetal gestational age and sex, pregnancy history, maternal medical history, and any known fetal genetic diagnoses. All cases were delivered in either the Special Delivery Unit at CHOP or at the University of Pennsylvania, and placentas were collected at delivery. Control subjects were provided shipment supplies for return of their placentas from delivery hospitals. Postnatal information was obtained from the medical record, or for controls, from a delivery information sheet with delivery course and neonatal biometrics filled out by the mother’s obstetrician. A fraction of controls were lost to follow-up and did not return their placentas and/or provide postnatal medical information. Controls received compensation of $25 for participation in and completion of the study.

### UVVF and combined cardiac output measurements

UVVF was calculated as previously published.^19^ Briefly, the umbilical vein was visualized in a free loop in the longitudinal plane and 3 measurements of the diameter of the vessel were averaged. Spectral Doppler of the umbilical vein was obtained at an angle of insonation of <20°. Due to the laminar flow pattern in the umbilical vein, the mean velocity is equal to half the maximum velocity. The cross-sectional area of the umbilical vein multiplied by the mean velocity of flow yields the rate of blood flow, UVVF, in mL/min. Interobserver reliability for UVVF measurements by this study’s investigators was previously reported as high,^19^ and these measurements are included in the standard fetal echocardiogram protocol at CHOP. Measurements were obtained at the time of the study but reviewed for adequacy and accuracy by R.J., D.Y.H., and Z.T.

Combined cardiac output (CCO) was assessed by multiplying the calculated area of both outflow tracts by the velocity-time integral determined by spectral Doppler for both the right and left ventricles and combining the values. In the case of single outflow tracts, only that value was used.

### Placental pathology and analysis

All placentas were examined in the pathology department at CHOP utilizing a systematic protocol, including recording the trimmed placental weight, membrane insertion, gross appearance, dimensions of the placental disc, and umbilical cord characteristics. Histologic samples included sections of membranes, umbilical cord, and at least 3 full-thickness sections of nonlesion placental parenchyma. Macroscopic and microscopic lesions were described according to the 2016 Amsterdam Placental Workshop Group^21^ and Freedman et al placental phenotypic classification systems^22^ (Supplemental Table 1). Microscopic examination was performed by a single perinatal pathologist for all placentas (R.L.), blinded to CHD type, UVVF values, and outcomes.

### Statistical analysis

Continuous variables are presented as median (IQR). Categorical parameters are described as frequency (N) and percentage (%). Controls were compared to all CHD and SV cases using chi-squared/Fisher’s tests and *t*-test/Wilcoxon tests depending on the variable type and distribution. *P* ≤ 0.05 was considered statistically significant. Linear and logistic regressions were performed to analyze the relationship between UVVF indexed to fetal weight (UVVF/Wt), placental defects, and outcomes. Following the exclusion of one patient with an outlier UVVF/Wt measurement, quantile regression was applied to: 1) assess the relationship between UVVF/Wt and gestational age; and 2) evaluate deviation from a healthy reference population. The optimal model for this study population was based on the Akaike information criterion and included both linear and quadratic terms.

All statistical analyses were conducted using R software, version 4.2.3 (R Development Core Team). R package rms was used for quantile regression and R package ggplot2 was used for figure generation.

## Results

### Subjects

A total of 38 fetal cases, the majority with SV CHD, as well as 36 fetal controls were included. The characteristics of the study population are shown in Table 1. The median gestational age of the cases was slightly higher than those of the controls (23 weeks vs 21 weeks; *P* < 0.001). Maternal age was slightly lower in cases compared to controls. There was no difference in the distribution of maternal race, comorbidities, medication exposure, or fetal sex between cases and controls. While only present in few subjects, there was no difference in prevalence of maternal hypertension, preeclampsia, or maternal aspirin use between groups. Simple, or class I maternal CHD^20^ was present in 4 controls (1 small VSD s/p spontaneous closure in childhood, 1 VSD s/p repair in childhood with no residual disease, 1 mild mitral valve prolapse, and 1 double aortic arch s/p neonatal repair), and in 1 case (small VSD s/p spontaneous closure). There was an increased prevalence of maternal diabetes in the control group, which may be due to diabetes being an indication for surveillance fetal echocardiography. One case was diagnosed postnatally with Rubinstein-Taybi syndrome. No other genetic anomalies were identified in cases or controls at the time of medical record review.

**Table 1:** Characteristics of the Study Population

	Controls (n = 36)	All CHD (n = 38)	*P* Value^a^	Single Ventricle (n = 28)	*P* Value^b^
Fetal gestational age (wk)	21.0 (20.0-22.0)	23.0 (22.3-26.8)^c^	**<0.001**	23.0 (22.8-25.0)^d^	**<0.001**
Maternal age (y)	32.0 (29.8-35.0)	30.0 (26.0-33.0)^e^	**0.022**	31.0 (27.0-34.0)	0.209
Maternal race			0.565		0.507
African American	3 (8.3%)	1/31 (3.2%)		0/22 (0%)	
Asian	3 (8.3%)	6/31 (19%)		4/22 (18%)	
Caucasian	28 (78%)	21/31 (68%)		16/22 (73%)	
Hispanic	1 (2.8%)	2/31 (6.5%)		1/22 (4.5%)	
Other	1 (2.8%)	1/31 (3.2%)		1/22 (4.5%)	
Fetal sex			0.532		0.539
Female	7/17 (41%)	12/37 (32%)		9 (32%)	
Male	10/17 (59%)	25/37 (68%)		19 (68%)	
Maternal comorbidity	19 (53%)	15 (39%)	0.251	12 (43%)	0.431
Chronic hypertension	3 (8.3%)	2 (5.3%)	0.670	2 (7.1%)	>0.999
Preeclampsia/Gestational hypertension	1 (2.8%)	3 (7.9%)	0.615	2 (7.1%)	0.577
Diabetes	10 (28%)	1 (2.6%)	**0.002**	0 (0%)	**0.003**
Autoimmune disease	0 (0%)	2 (5.3%)	0.494	2 (7.1%)	0.188
Maternal CHD	4 (11%)	1 (2.6%)	0.194	1 (3.6%)	0.375
Obesity	0 (0%)	2 (5.3%)	0.494	2 (7.1%)	0.188
Renal disease	0 (0%)	0 (0%)		0 (0%)	
Smoker	0 (0%)	0 (0%)		0 (0%)	
Aspirin use	5 (14%)	3 (7.9%)	0.474	2 (7.1%)	0.454
Antihypertensive medication use	0 (0%)	1 (2.6%)	>0.999	1 (3.6%)	0.438

CHD = congenital heart disease.

Values are median (IQR) or n (%). **Bold** text indicates *P* ≤ 0.05.

a *P* value comparison between all CHD subjects and controls.

b *P* value comparison between single ventricle subjects and controls.

c n = 31 for fetal gestational age for all CHD subjects.

d n = 24 for fetal gestational age for single ventricle subjects.

e n = 37 for maternal age for all CHD subjects.

### Placental blood flow

**Figure 1:**
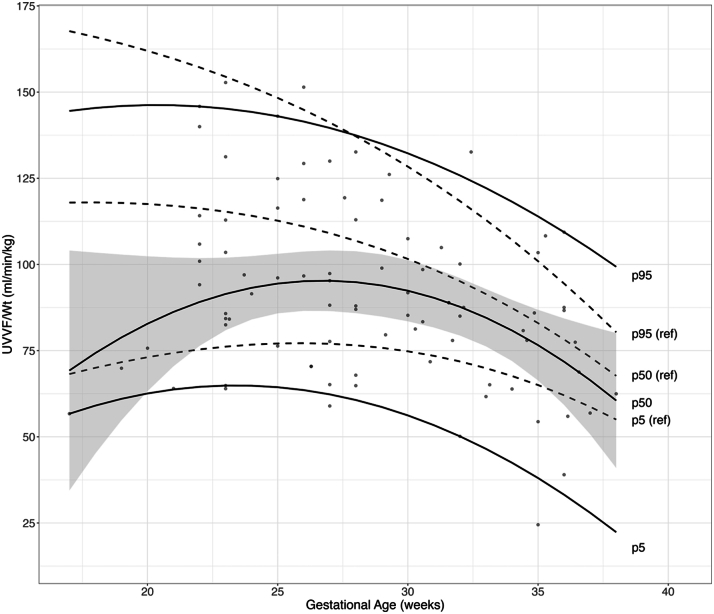
**Umbilical Venous Volume Flow/Wt Throughout Gestation in Congenital Heart Disease Fetuses** UVVF/Wt (ml/min/kg) was measured from serial fetal echocardiograms across gestational age (GA) in CHD fetuses. 95% (p95), 50% (p50), and 5% (p5) are represented by solid lines using a quantile regression with linear and quadratic terms. p5 = 9.35∗GA - 0.20∗GA^2^ - 44.54. p50 = 14.68∗GA – 0.27∗GA^2^ - 100.97. p95 = 6.14∗GA – 0.15∗GA^2^ +83.73. Gray shading represents 95% confidence interval of the p50. Gray dashed lines represent the previously published 5% (p5 [ref]), 50% (p50 [ref]), and 95% (p95 [ref]) regression model of UVVF/Wt over gestation in healthy fetuses.^23^ Average UVVF/Wt for cases was decreased compared to the reference 50th percentile for healthy fetuses in the 2nd trimester, however continued to trend slightly lower than normal in later gestation. CHD = congenital heart disease; UVVF = umbilical venous volume flow.

On manual review, 7 cases lacked adequate image quality for an accurate calculation of second trimester UVVF. At the time of fetal echocardiography, the median fetal weight for cases was higher than controls, in the setting of slightly higher median gestational age (Table 2). Accordingly, absolute UVVF was not different between cases and controls. When UVVF was indexed to fetal weight (UVVF/Wt), it was significantly decreased in all cases, as well as the SV subgroup, compared to controls. UVVF as a proportion of cardiac output (UVVF/CCO), reflecting the proportion of fetal circulation returning to the placenta, was significantly decreased in all cases, as well as the SV subgroup, compared to controls. While still within the normal range, there was an increased mean UA pulsatility index (UA PI), and a decreased mean uterine artery PI (UtA PI) in cases compared to controls. The middle cerebral artery PI (MCA PI) and CPR were not different (Table 2). UVVF/Wt slowly declined throughout the late 2nd trimester and 3rd trimester in cases who underwent at least one follow-up study (n = 23) (Figure 1). Compared to published norms,^23^ average UVVF/Wt values for cases were decreased compared to the 50th percentile for healthy fetuses in the 2nd trimester, however continued to trend slightly lower than normal in later gestation.

**Table 2:** Placental Blood Flow Characteristics

	Controls (n = 36)	All CHD (n = 31)	*P* Value^a^	Single Ventricle (n = 24)	*P* Value^b^
Fetal weight (g)	437 (340-546)	629 (530-756)	**<0.001**	646 (528-830)	**<0.001**
CCO (mL/min/kg)	394 (345-472)^c^	442 (321-542)	0.574	371 (321-471)	0.555
UVVF (mL/min)	51 (38-68)	61 (47-84)	0.147	59 (44-80)	0.333
UVVF/Wt (mL/min/kg)	113 (98-145)	96 (79-115)	**0.007**	87 (74-108)	**0.001**
UVVF/CCO (%)	30 (24-39)^c^	22 (18-30)	**0.006**	23 (20-31)	**0.045**
MCA PI	1.67 (1.55-1.79)^c^	1.78 (1.55-1.93)	0.261	1.77 (1.49-1.89)	0.660
UA PI	1.23 (1.16-1.36)	1.36 (1.27-1.48)	**0.020**	1.33 (1.28-1.39)	0.091
UTA PI	0.96 (0.78-1.24)	0.78 (0.66-1.00)	**0.017**	0.80 (0.67-0.99)	**0.038**
CPR	1.36 (1.17-1.52)^c^	1.29 (1.10-1.50)	0.404	1.30 (1.14-1.46)	0.413

Values are median (IQR). **Bold** text indicates *P* ≤ 0.05.

CCO = combined cardiac output; CPR = cerebroplacental ratio (MCA/UA); MCA = middle cerebral artery; PI = pulsatility index; UA = umbilical artery; UTA = uterine artery; UVVF = umbilical venous volume flow; other abbreviation as in Table 1.

a *P* value comparison between all CHD subjects and controls.

b *P* value comparison between single ventricle subjects and controls.

c n = 35 for CCO, UVVF/CCO, MCA PI, and CPR for controls.

### Placental pathology

Thirty-nine percent of control subjects were lost to follow-up and did not provide their placenta or postnatal data for analysis. Placentas from 2 cases who delivered at an outside institution were not available for analysis. In those analyzed, there was no significant difference between placental weight in cases compared to controls (Table 3). There was also no difference in the ratio of placental weight to birth weight (PW:BW), an indicator of placental efficiency. Notably, 44% of all cases and 41% of SV cases had lesions associated with maternal vascular malperfusion (MVM) compared to 18% of controls (*P* = 0.041, *P* = 0.088). In particular, cases had significantly more high-grade MVM lesions compared to controls (39% cases vs 9.1% controls, *P* = 0.049). Lesions associated with fetal vascular malperfusion (FVM) were found in 42% of total cases, and 41% of SV cases, compared to 23% of controls, however this finding was not statistically significant (*P* = 0.141, *P* = 0.181). There was no difference in the prevalence of other placental abnormalities between groups, including umbilical cord abnormalities, chronic inflammation, increased villous vascularity, or other significant pathology (massive perivillous fibrin deposition, delayed villous maturation, or chorangiosis). There was a nonsignificant trend toward an increased prevalence of acute inflammatory lesions in the control placentas (*P* = 0.055).

**Table 3:** Placental Pathology in Cases Vs Controls

	Controls (n = 22)	All CHD (n = 36)	*P* Value^a^	Single Ventricle (n = 27)	*P* Value^b^
Placenta weight (g)	442 (428-473)	426 (332-490)	0.332	412 (335-493)	0.300
PW:BW	0.13 (0.12-0.14)^c^	0.13 (0.11-0.15)	0.820	0.12 (0.11-0.15)	0.574
Cord abnormality	6 (27%)	11 (31%)	0.790	5 (19%)	0.510
Acute inflammation (AI)	13 (59%)	12 (33%)	0.055	11 (41%)	0.201
AI grade			0.115		0.376
0	9 (41%)	24 (67%)		16 (59%)	
1	11 (50%)	9 (25%)		8 (30%)	
2	2 (9.1%)	3 (8.3%)		3 (11%)	
Chronic inflammation (CI)	10 (45%)	23 (64%)	0.169	17 (63%)	0.220
CI grade			0.382		0.444
0	12 (55%)	13 (36%)		10 (37%)	
1	6 (27%)	13 (36%)		9 (33%)	
2	4 (18%)	10 (28%)		8 (30%)	
Maternal vascular malperfusion (MVM)	4 (18%)	16 (44%)	**0.041**	11 (41%)	0.088
MVM grade			**0.049**		0.063
0	18 (82%)	20 (56%)		16 (59%)	
1	2 (9.1%)	2 (5.6%)		1 (3.7%)	
2	2 (9.1%)	14 (39%)		10 (37%)	
Fetal vascular malperfusion (FVM)	5 (23%)	15 (42%)	0.141	11 (41%)	0.181
FVM grade			0.295		0.449
0	17 (77%)	21 (58%)		16 (59%)	
1	3 (14%)	11 (31%)		8 (30%)	
2	2 (9.1%)	4 (11%)		3 (11%)	
Increased villous vascularity	3 (14%)	6 (17%)	>0.999	5 (19%)	0.715
Other pathologies^d^	7 (32%)	14 (39%)	0.587	13 (48%)	0.247

PW:BW = placental weight: birth weight ratio; other abbreviation as in Table 1.

Values are median (IQR) or n (%). **Bold** text indicates *P* ≤ 0.05.

a *P* value comparison between all CHD subjects and controls.

b *P* value comparison between single ventricle subjects and controls.

c n = 18 for PW:BW for controls samples.

d Other pathologies include presence of hemosiderosis, massive perivillous fibrin deposition, delayed villous maturation, chorangiosis, or isolated small for gestational age placenta (<10th percentile).

### Clinical outcomes

There was no difference in birth gestational age between cases and controls (Table 4). All cases, as well as the SV subgroup, demonstrated significantly decreased birth weight compared to controls (*P* = 0.026 for all CHD, *P* = 0.050 for SV). Birth length was also decreased in cases however there was significant attrition (75%) due to loss to follow-up in controls for this outcome (Table 4). There was 1 intrauterine fetal demise at term in a case. All other cases survived to 30 days if inpatient or index hospital discharge, whichever came first.

**Table 4:** Clinical Outcomes in Cases Vs Controls

	Controls (n = 22)	All CHD (n = 36)	*P* Value^a^	Single Ventricle (n = 27)	*P* Value^b^
Gestational age at delivery (wk)	39.2 (38.5-39.6)^c^	38.9 (38.3-39.6)	0.236	39.0 (38.3-39.7)	0.437
Birth weight (g)	3,358 (3,165-3,742)^c^	3,145 (2,873-3,462)	**0.026**	3,160 (2,905-3,502)	**0.050**
Birth length (cm)	51.4 (49.5-52.5)^c^	48.4 (47.1-50.0)^d^	0.012	48.9 (48.0-51.0)^e^	0.048
Head circumference (cm)	35.0 (33.8-35.5)^c^	33.5 (32.5-34.5)^d^	0.086	33.5 (32.5-34.5)^e^	0.177

Abbreviation as in Table 1.

Values are median (IQR). **Bold** text indicates *P* ≤ 0.05.

a *P* value comparison between all CHD subjects and controls.

b *P* value comparison between single ventricle subjects and controls.

c Controls: n = 18 for gestational age at delivery and birth weight. n = 9 for birth length. n = 8 for head circumference.

d All CHD: n = 34 for birth length. n = 33 for head circumference.

e Single ventricle: n = 25 for birth length and head circumference.

**Figure 2:**
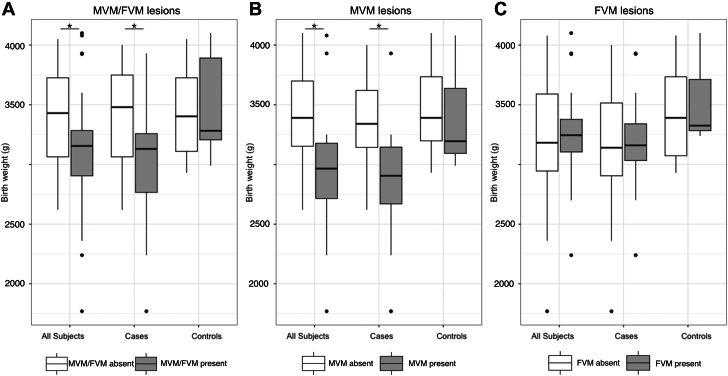
**Impaired Fetal Growth in Congenital Heart Disease Occurs Exclusively With Maternal Vascular Malperfusion Lesions** (A) CHD fetuses but not controls demonstrate significantly decreased birth weight (3,480 g vs 3,130 g; *P* = 0.04) in the presence of PMP lesions. Decreased birth weight in CHD fetuses is seen only in the presence of MVM lesions (3,340 g vs 2,905 g; *P* < 0.001) (B), and not FVM lesions (3,140 g vs 3,160 g; *P* = 0.7) (C). FVM = fetal vascular malperfusion; MVM = maternal vascular malperfusion; PMP = placental malperfusion; other abbreviation as in Figure 1.

When comparing fetuses with evidence of either MVM or FVM to those without, there was no significant difference in the mid-gestational UVVF/Wt of cases or controls (Table 5). However, there was significantly decreased birth weight in cases with evidence of either MVM or FVM compared to cases without, which was not present in the control group, though in the setting of 39% attrition of controls for this outcome. Univariable linear regression demonstrated that for cases, the presence of either MVM or FVM lesions was associated with a 382g decrease in birth weight (*P* = 0.029), a relationship not seen in the control group (*P* = 0.692) (Table 6). Decreased birth weight in cases compared to controls appeared to be related to MVM lesions in particular but was independent of the presence of FVM lesions (Figure 2). Accordingly, the presence of MVM lesions was associated with a 531g decrease in birth weight for all CHD (*P* < 0.001), and 403g decrease in birth weight for SV cases (*P* = 0.017). There was no significant relationship between MVM lesions and birth weight in the control group, or between FVM lesions and birth weight for any subjects (Table 6). There was no significant difference in median intensive care unit length of stay (ICU LOS) in cases with evidence of either MVM or FVM compared to cases without (Table 5). Regression analyses did not demonstrate a significant relationship between mid-gestational UVVF/Wt and clinical outcomes (data not shown).

**Table 5:** The Influence of Placental Malperfusion Defects on UVVF and Outcomes

	Controls			All CHD		
	MVM/FVM Absent	MVM/FVM Present	*P* Value	MVM/FVM Absent	MVM/FVM Present	*P* Value
	(n = 11)	(n = 9)		(n = 11)	(n = 18)	
UVVF/Wt (ml/min/kg)	99.7 (93.2-133.5)	119.1 (113.1-161.7)	0.056	88.2 (83.4-106.9)	96.7 (71.3-118.2)	0.982

	(n = 12)	(n = 6)		(n = 12)	(n = 24)	
Birth weight (g)	3,402 (3,110-3,726)	3,282 (3,206-3,891)	0.750	3,480 (3,064-3,749)	3,130 (2,766-3,258)	**0.039**

				(n = 12)	(n = 23)	
ICU LOS (d)				14.5 (10.0-20.5)	17 (10.3-30.0)	0.444

Values are median (IQR). **Bold** text indicates *P* ≤ 0.05.

FVM = fetal vascular malperfusion; ICU LOS = index hospitalization ICU length of stay; MVM = maternal vascular malperfusion; other abbreviations as in Tables 1 and 2.

**Table 6:** Univariable Linear Regression Modeling of the Association Between Presence of Placental Malperfusion Lesions and Birth Weight (g)

	Controls (n = 18)	*P* Value	All CHD (n = 36)	*P* Value	Single Ventricle (n = 27)	*P* Value
Predictor						
MVM/FVM	81.42 (−346.70 to 509.53)	0.692	−381.75 (−726.59 to −36.91)	**0.031**	−230.00 (−599.73 to 139.73)	0.212
MVM	−14.87 (−559.08 to 529.35)	0.955	−531.56 (−829.27 to −233.85)	**<0.001**	−403.47 (−729.76 to −77.17)	**0.017**
FVM	145.13 (−393.67 to 683.94)	0.576	62.34 (−290.41 to 415.10)	0.722	189.46 (−168.31 to 547.23)	0.286

Values are beta coefficient and (95% CI). Three separate univariable linear regressions were applied to each patient population. The independent variable was presence of MVM/FVM lesions, presence of MVM lesions, or presence of FVM lesions. For all models, birth weight (g) was the dependent variable. **Bold** text indicates *P* ≤ 0.05.

Abbreviations as in Tables 1 and 5.

## Discussion

We previously demonstrated in a retrospective study that fetuses with CHD have decreased mid-gestational placental blood flow, however correlation with placental pathology could not be performed.^19^ In this prospective cohort, we demonstrate that CHD fetuses, specifically those with SV physiology, have decreased mid-gestational placental blood flow, a high prevalence of placental malperfusion (PMP) defects, and impaired fetal growth resulting in lower birth weight at similar gestational age compared to normal fetuses (see Central Illustration). CHD fetuses had on average only 22%, rather than the normal 30%, of fetal CCO returning from the placenta, which we hypothesize, may be due to increased placental resistance. The effect of redistribution of fetal blood flow is unclear, as cerebral blood flow reflected by absolute mid-gestational CPR was not different. Assessment of middle cerebral artery PI and CPR *z*-scores may better reflect the distribution of fetal blood flow given varying fetal gestational ages. The increased mid-gestational UA PI in cases may also reflect increased placental resistance, however decreased flow through the UA despite increased placental resistance may normalize the UA PI, and thus UVVF may be a more reliable indicator of placental flow. The significance of decreased UtA PI in cases is unclear but suggests that placental resistance was not sufficiently elevated at mid-gestation to result in elevated UtA Doppler parameters. Our work confirms that abnormal placental blood flow in fetuses with CHD can be detected as early as mid-gestation.

**Central Illustration:**
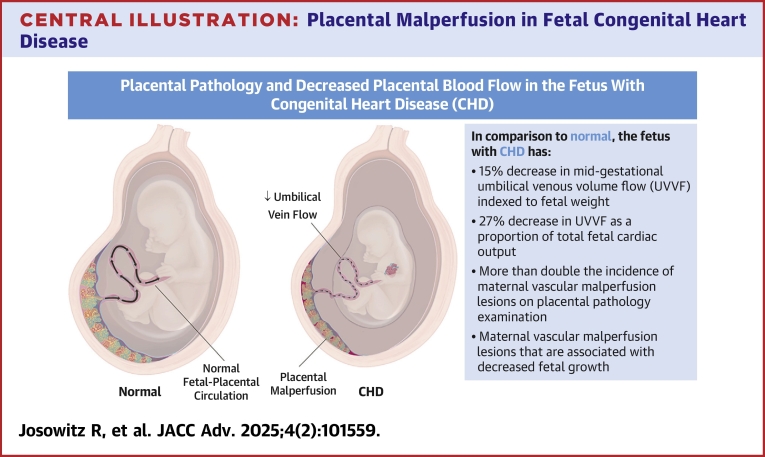
**Placental Malperfusion in Fetal Congenital Heart Disease** Fetuses with CHD have decreased blood flow from the placenta to fetus through the umbilical vein, as measured in second trimester fetal echocardiograms by umbilical venous volume flow. In the setting of unchanged cardiac output, decreased umbilical flow is suggestive of increased placental resistance. These fetuses have a higher incidence of placental malperfusion lesions at birth. Placental maternal vascular malperfusion lesions, in particular, are associated with decreased fetal growth in CHD. Abbreviation as in Figure 1.

We have undertaken a rigorous analysis of placental pathology in CHD based on standardized placental phenotypic classification systems.^21^^,^^22^ An increased frequency of MVM and FVM defects has been reported in CHD.^24^, ^25^, ^26^ The frequency of our placental findings in CHD is similar to published reports,^26^^,^^27^ however reported frequencies vary widely due to variable patient and control populations, and criteria for placental pathologic diagnosis. FVM can occur secondary to umbilical cord obstruction or flow alterations in CHD which could result in decreased placental blood flow and stasis.^28^ MVM is due to defective placental implantation in early pregnancy resulting in inadequate maternal spiral artery remodeling and disrupted intervillous flow.^25^ We did not find a significant difference in umbilical cord abnormalities, as has been previously reported in CHD,^29^^,^^30^ or in the frequency of chronic inflammatory lesions, which when severe, can be a cause of FVM.^21^ The trend toward increased low-grade acute inflammation in controls likely reflects induction of most CHD mothers prior to the onset of natural labor, while most control mothers labored naturally.^31^

Interestingly, decreased birth weight in cases compared to controls was only observed in the presence of MVM lesions but was independent of FVM lesions. MVM lesions in controls were not associated with decreased birth weight in our cohort though the number of control samples with MVM was small, suggesting a possible multifactorial etiology for decreased birth weight in CHD. No change in birth weight was seen in the setting of FVM lesions for cases or controls. MVM and FVM lesions are associated with intrauterine growth restriction,^25^^,^^28^ a common finding in CHD.^24^, ^25^, ^26^, ^27^^,^^32^ Some retrospective studies lacking control groups have not implicated other placental pathologies in low birth weight in CHD,^33^^,^^34^ however low placental weight *z*-score at birth and low placental volume by magnetic resonance imaging have been associated with decreased fetal growth in CHD in other studies.^5^^,^^35^ A direct association specifically between PMP and birth weight in CHD is rarely interrogated.^33^^,^^36^^,^^37^ Our data suggest that the mechanisms underlying MVM may act in synergy with the impaired fetal hemodynamics and/or alterations in systemic oxygenation in fetal CHD to impair fetal growth. While we had hypothesized that UVVF might be a mid-gestational marker of PMP, our analyses revealed no significant association between mid-gestational UVVF and placental pathologic findings or measures of fetal growth. Nevertheless, we demonstrate tangible evidence of altered fetal-placental circulation in CHD. Better understanding of the role of abnormal placental blood flow in CHD could inform the future use of therapeutic agents in utero such as aspirin, known to improve placental perfusion in maternal preeclampsia.^38^

There are several limitations to this study. The small sample size resulted in the study likely being underpowered to achieve statistical significance where clinically meaningful differences may exist. The study may have been underpowered to determine the nature of the relationship between UVVF, placental abnormalities, and many of our study outcomes. Due to technical issues or loss to follow-up, some data were not available for all subjects. High rates of attrition for control subjects’ placental and outcomes data could introduce bias, affecting the validity of these outcomes. As UVVF was measured at mid-gestation, there may be additional factors not accounted for that affect placental development between mid-gestation and delivery. Our study demonstrated several significant findings despite a number of factors which may have biased this study toward the null hypothesis. Control subjects were recruited from referrals due to clinical indications for screening fetal echocardiograms, and thus do not fully represent low risk, normal pregnancies. Eleven cases may have received vaginal progesterone (randomization was blinded) as part of an unrelated clinical trial evaluating the effect of progesterone on neurodevelopment in CHD, an outcome not analyzed in our study. Vaginal progesterone has been associated with vasodilation and improved Doppler flow parameters in utero,^39^ which could have biased our results toward the null hypothesis with regard to UVVF. Its impact on PMP lesions at birth is unknown. The frequency of placental abnormalities in our prospective control population may be higher than in more commonly used retrospective control populations, as several indications for fetal echocardiography (ie, diabetes) may increase the risk of abnormal placental pathology. Similarly, the presence of maternal CHD, present in 4 controls and 1 case, has been associated with PMP and decreased birth weight. However, when stratified based on CHD severity according to the 2018 American Heart Association/American College of Cardiology guidelines,^20^ several studies have not found a robust association between “simple” maternal CHD, similar to that found in our study subjects, and PMP or impaired fetal growth.^40^^,^^41^ Comparison of our control subjects with and without both maternal diabetes and CHD revealed no significant differences in maternal or fetal characteristics, placental blood flow, placental pathology, or outcomes (Supplemental Tables 2 to 5). Any predisposition to placental defects or poor growth due to maternal diabetes or CHD would most likely bias our results toward the null hypothesis; however, we were able to detect meaningful comparative increases in placental pathology in our cohort. Our findings are most relevant to fetuses with SV CHD and may not be generalizable to other CHD subgroups. Future work comparing placental blood flow and pathology between right and left-sided obstructive lesions will be of great interest, as the degree of fetal brain and placental hypoxia varies.^42^ Additional studies in a larger cohort are necessary to determine the relationship between abnormal UVVF parameters and placental pathology at birth, and whether they are associated with additional outcomes of interest such as abnormal neurodevelopment and increased mortality, which have been associated with placental abnormalities in CHD.^2^^,^^43^

## Conclusions

In this prospective study, we demonstrate that fetuses with CHD, specifically SV CHD, have decreased mid-gestational placental blood flow, increased frequency of PMP defects, and impaired fetal growth. In addition, a rigorous and systematic evaluation of the placenta in fetal CHD demonstrates an association between decreased fetal growth and the presence of placental MVM lesions but not FVM lesions. Our study did not demonstrate significant associations between impaired mid-gestational UVVF and placental pathologic findings. Despite this, decreased UVVF reflects tangible evidence of abnormal in utero fetal placental blood flow in CHD. There remain significant unknowns regarding the abnormal fetal circulation, its relationship to placental abnormalities in CHD, and how they may contribute to outcome disparities which warrant future investigation. Understanding the mechanism underlying PMP in CHD is of great interest and could inform novel therapeutic targets.

Perspectives **COMPETENCY IN MEDICAL KNOWLEDGE:** CHD fetuses have altered fetal-placental circulation detectable as early as mid-gestation by decreased UVVF, and a high prevalence of PMP. The presence of MVM is associated with decreased birth weight in CHD.

**TRANSLATIONAL OUTLOOK:** Early detection of abnormal placental perfusion in CHD fetuses could inform the evaluation of potential innovative therapeutic interventions to improve placental perfusion in utero. Additional studies in larger cohorts are necessary to determine the relationship between UVVF, placental pathology, and outcomes.

## Funding support and author disclosures

This work was supported by a Children’s Hospital of Philadelphia Cardiac Center Research Grant and the Robert & Dolores Harrington Endowed Chair in Cardiology at the Children’s Hospital of Philadelphia. The authors have reported that they have no relationships relevant to the contents of this paper to disclose.
