# Supplementary material for: Congenital Heart Disease Fetuses Have Decreased Mid-Gestational Placental Flow, Placental Malperfusion Defects, and Impaired Growth
Source: JACC Adv. 2025 Jan 16;4(2):101559. doi: 10.1016/j.jacadv.2024.101559 (PMC11786062; doi:10.1016/j.jacadv.2024.101559)
Supplement: Supporting information [file mmc1.docx]

Supplemental Table 1. Definitions of placental pathological lesions, based on consensus classification systems^24,25^.

| **Placental pathology variable** | **Definition** |
| --- | --- |
| Placental weight (g) | Trimmed placental weight (grams) |
| PW:BW | Trimmed Placental weight (g) : Birth weight(g) ratio |
| Cord abnormality | Presence of hypocoiled umbilical cord (<0.1 coils/cm), hypercoiled umbilical cord (>0.3 coils/cm)2, marginal insertion of umbilical cord, velamentous insertion of umbilical cord, two vessel umbilical cord, true knot of umbilical cord, or long umbilical cord (>70 cm in length) |
| Acute inflammation (AI) | Presence of any of the following:   - Maternal: acute subchorionitis, chorionitis, amnionitis or necrotizing chorioamnionitis - Fetal: acute chorionic vasculitis, phlebitis, arteritis/panvasculitis, funisitis |
| AI grade | 0 – no acute inflammation  1 – low grade – stage 1 maternal and/or fetal inflammatory response based on Amsterdam consensus statement  2 – high grade – stage 2 or 3 maternal and/or fetal inflammatory response based on Amsterdam consensus statement |
| Chronic inflammation (CI) | Presence of CI in any of the following compartments:   - Membranes/chorionic plate: chronic chorionitis or amnionitis - Basal plate: chronic deciduitis with plasma cells, chronic decidual perivasculitis - Villi: chronic villitis, intravillous plasma cells - Intervillous space: chronic intervillositis - Fetal: chronic eosinophilic/T-cell vasculitis, chronic fetal inflammation |
| CI grade | 0 – no chronic inflammation  1 – 1 compartment with chronic inflammation  2 – 2 or more compartments with chronic inflammation |
| Fetal vascular malperfusion (FVM) | Presence of thrombi or intramural fibrin deposition in chorionic vessels, velamentous vessels, stem villous vessels, and or umbilical vessels; avascular villi or villous stromal vascular karyorrhexis - At least 3 foci of avascular villi and/or villous stromal vascular karyorrhexis (≥ 3 villi/focus) |
| FVM grade | 0 – no FVM lesions  1 – any FVM lesions no indicated as high grade (2)  2 – high grade FVM defined as ≥ 2 fetal chorionic or stem villous vascular thrombi or a total of ≥ 45 avascular villi over 3 sections examination |
| Maternal vascular malperfusion (MVM) | Presence of any of the following (1 point)   - Decidual vasculopathy (fibrinoid necrosis/acute atherosis, muscularization of basal plate arterioles, mural hypertrophy of membrane arterioles, basal decidual vascular thrombus) (24) - Single villous infarction - Accelerated villous maturation (increased syncytial knots, villous agglutination, increased perivillous fibrin deposition) (24) - Distal villous hypoplasia (24) - Changes consistent with retroplacental blood/hematoma   Presence of any of the following (2 points)   - Multiple infarcts - Retroplacental hematoma with hemosiderin or infarct - Placental hypoplasia (<10^th^ percentile placental weight) in the presence of 1 or more maternal vascular lesions (at least 1 point) |
| MVM grade | 0 – none – score of 0 or 1 points  1 – low grade – score of 2-3 points  2 – high grade – score of ≥ 4 points |
| Increased villous vascularity | Regionally increased villous vascularity insufficient for the diagnosis of villous chorangiosis  Villous chorangiosis  Villous chorangiomatosis |
| Other pathologies | Presence of hemosiderosis, massive perivillous fibrin deposition, delayed villous maturation, chorangiosis, or isolated small for gestational age placenta (<10^th^ percentile) |

Supplemental Table 2. Comparison of maternal and fetal characteristics between controls with and without maternal CHD and diabetes.

| **Characteristic** | **Controls without maternal CHD/Diabetes**  **(n = 22)** | **Controls with maternal CHD/Diabetes**  **(n = 14)** | **p** |
| --- | --- | --- | --- |
| **Gestational Age (weeks)** | 20.50 (19.00, 22.00) | 21.00 (21.00, 24.00) | 0.059 |
| **Maternal Age** | 32.0 (30.0, 34.0) | 33.5 (29.0, 36.0) | 0.557 |
| **Race** |  |  | 0.068 |
| African American | 0 (0%) | 3 (21%) |  |
| Asian | 2 (9.1%) | 1 (7.1%) |  |
| Caucasian | 19 (86%) | 9 (64%) |  |
| Hispanic | 1 (4.5%) | 0 (0%) |  |
| Other | 0 (0%) | 1 (7.1%) |  |
| **Fetal Gender** |  |  | 0.250 |
| Female | 4/13 (31%) | 3/4 (75%) |  |
| Male | 9/13 (69%) | 1/4 (25%) |  |
| **Maternal Comorbidity** |  |  |  |
| **Chronic Hypertension** | 2 (9.1%) | 1 (7.1%) | >0.999 |
| **Preeclampsia/Gestational Hypertension** | 1 (4.5%) | 0 (0%) | >0.999 |
| **Diabetes** | 0 (0%) | 10 (71%) | **<0.001** |
| **Maternal CHD** | 0 (0%) | 4 (29%) | **0.017** |
| **Aspirin use** | 3 (14%) | 2 (14%) | >0.999 |

Data presented as n (%) of study subjects or median (IQR) for continuous variables.

Supplemental Table 3. Comparison of placental blood flow characteristics between controls with and without maternal CHD and diabetes.

| **Characteristic** | **Controls without maternal CHD/Diabetes**  **(n = 22)** | **Controls with maternal CHD/Diabetes**  **(n = 14)** | **p** |
| --- | --- | --- | --- |
| **Fetal Weight (g)** | 391 (317, 522) | 479 (427, 667) | 0.083 |
| **CCO (ml/min/kg)** | 416 (319, 481) | 389 (355, 428)^a^ | 0.625 |
| **UVVF (ml/min)** | 50 (36, 60) | 60 (40, 101) | 0.191 |
| **UVVF/Wt (ml/min/kg)** | 108 (96, 132) | 122 (100, 158) | 0.470 |
| **UVVF/CCO (%)** | 28 (23, 39) | 32 (27, 41)^a^ | 0.448 |
| **MCA PI** | 1.67 (1.50, 1.75) | 1.67 (1.58, 2.00)^a^ | 0.297 |
| **UA PI** | 1.26 (1.17, 1.39) | 1.24 (1.15, 1.29) | 0.337 |
| **UTA PI** | 1.02 (0.73, 1.25) | 0.91 (0.78, 1.09) | 0.548 |
| **CPR** | 1.30 (1.15, 1.47) | 1.46 (1.27, 1.74)^a^ | 0.116 |

Data presented as median (IQR). ^a^ n=13 for CCO, UVVF/CCO, MPA PI, and CPR for controls with maternal CHD/diabetes. CCO, combined cardiac output; CPR, cerebroplacental ratio (MCA/UA); MCA, middle cerebral artery; PI, pulsatility index; UA, umbilical artery; UTA, uterine artery; UVVF, umbilical venous volume flow.

Supplemental Table 4. Comparison of placental pathology between controls with and without maternal CHD and diabetes.

| **Characteristic** | **Controls without maternal CHD/Diabetes**  **(n = 15)** | **Controls with maternal CHD/Diabetes**  **(n = 7)** | **p** |
| --- | --- | --- | --- |
| **Placenta Weight (g)** | 440 (404, 470) | 442 (436, 578) | 0.397 |
| **PW:BW** | 0.124 (0.115, 0.134)^a^ | 0.141 (0.125, 0.151)^b^ | 0.233 |
| **Cord Abnormality** | 5 (33%) | 1 (14%) | 0.616 |
| **Acute Inflammation (AI)** | 11 (73%) | 2 (29%) | 0.074 |
| AI Grade |  |  | 0.186 |
| 0 | 4 (27%) | 5 (71%) |  |
| 1 | 9 (60%) | 2 (29%) |  |
| 2 | 2 (13%) | 0 (0%) |  |
| **Chronic Inflammation (CI)** | 6 (40%) | 4 (57%) | 0.652 |
| CI Grade |  |  | 0.826 |
| 0 | 9 (60%) | 3 (43%) |  |
| 1 | 4 (27%) | 2 (29%) |  |
| 2 | 2 (13%) | 2 (29%) |  |
| **Maternal Vascular Malperfusion (MVM)** | 3 (20%) | 1 (14%) | >0.999 |
| MVM Grade |  |  | >0.999 |
| 0 | 12 (80%) | 6 (86%) |  |
| 1 | 1 (6.7%) | 1 (14%) |  |
| 2 | 2 (13%) | 0 (0%) |  |
| **Fetal Vascular Malperfusion (FVM)** | 2 (13%) | 3 (43%) | 0.274 |
| FVM Grade |  |  | 0.197 |
| 0 | 13 (87%) | 4 (57%) |  |
| 1 | 1 (6.7%) | 2 (29%) |  |
| 2 | 1 (6.7%) | 1 (14%) |  |
| **Increased Villous Vascularity** | 1 (6.7%) | 2 (29%) | 0.227 |
| **Other Pathologies^c^** | 5 (33%) | 2 (29%) | >0.999 |

Data presented as median (IQR). ^a,b^ n=14 and n=4 for PW:BW for respective groups. ^c^ Other pathologies include presence of hemosiderosis, massive perivillous fibrin deposition, delayed villous maturation, chorangiosis, or isolated small for gestational age placenta (<10^th^ percentile). AI, acute inflammation; CI, chronic inflammation; FVM, fetal vascular malperfusion; MVM, maternal vascular malperfusion; PW:BW, placental weight: birth weight ratio.

Supplemental Table 5. Comparison of clinical outcomes between controls with and without maternal CHD and diabetes.

| **Characteristic** | **Controls without CHD/Diabetes**  **(n = 15)** | **Controls with CHD/Diabetes**  **(n = 7)** | **p** |
| --- | --- | --- | --- |
| **Gestational Age at Delivery** **(weeks)** | 39.4 (39.0, 39.9)^a^ | 38.6 (38.1, 39.2)^b^ | 0.263 |
| **Birth weight (g)** | 3,315 (2,990, 3,750)^a^ | 3,522 (3,323 3,909)^b^ | 0.327 |
| **Birth length (cm)** | 51.1 (49.8, 51.9)^c^ | 52.5 (50.5, 53.0)^e^ | 0.548 |
| **Head circumference (cm)** | 35.0 (33.0, 35.5)^d^ | 35.0 (34.5, 35.8)^e^ | 0.651 |

Data presented as median (IQR). ^a^ Controls without CHD/diabetes: n=14 for gestational age and birth weight. ^b^ Controls with CHD/diabetes: n= 4 for gestational age and birth weight. ^c-d^ Controls without CHD/diabetes: n=6 for birth length, n=5 for head circumference. ^e^ Controls with CHD/diabetes: n= 3 for birth length and head circumference.
